# Blood Culture Positivity, Microbiological Spectrum, and Inflammatory Correlates in Patients Hospitalized with SARS-CoV-2 Infection During the Omicron BA.5 Wave: A Retrospective Cohort Study

**DOI:** 10.3390/microorganisms14091941

**Published:** 2026-09-02

**Authors:** Cristiana Georgeta Bujor, Marilena Dinuti, Felicia Sfrijan

**Affiliations:** Department IV—Biochemistry and Pharmacology, Faculty of Medicine, “Victor Babeş” University of Medicine and Pharmacy Timișoara, Eftimie Murgu Square 2, 300041 Timișoara, Romania; bujor.cristiana@umft.ro (C.G.B.); dinuti.marilena@umft.ro (M.D.)

**Keywords:** SARS-CoV-2, COVID-19, Omicron BA.5, blood culture, bacteremia, bloodstream infection, bacterial coinfection, sepsis, antimicrobial stewardship

## Abstract

Blood cultures are frequently obtained in hospitalized patients with COVID-19, although the diagnostic yield and clinical meaning of culture positivity vary markedly according to case mix, disease severity, and timing of sampling. Omicron-era data remain comparatively limited. We aimed to characterize blood-culture positivity, pathogen distribution, inflammatory correlates, antimicrobial exposure, and short-term outcomes in a homogeneous cohort hospitalized during the SARS-CoV-2 Omicron BA.5 wave. This retrospective observational study included 395 patients hospitalized with SARS-CoV-2 infection between 12 July and 25 September 2022. Patients were categorized as blood-culture positive when a named bacterial species was recorded and as culture negative when “No growth” was documented. Continuous variables were compared using the Mann–Whitney U test and categorical variables using Fisher’s exact or chi-square tests, as appropriate. A parsimonious multivariable logistic regression model was used to explore factors independently associated with blood-culture positivity. Blood cultures were positive in 89/395 patients (22.5%). Gram-positive organisms accounted for 52.8% of positive cultures and Gram-negative organisms for 47.2%. The most frequent isolates were *Streptococcus pneumoniae* (20.2% of positive cultures), *Enterococcus faecalis* (18.0%), *Klebsiella pneumoniae* (18.0%), *Staphylococcus aureus* (14.6%), *Escherichia coli* (14.6%), and *Pseudomonas aeruginosa* (14.6%). Compared with patients with no growth, those with positive cultures had higher admission leukocyte counts (median 13.42 vs. 11.22 × 10^9^/L; *p* < 0.001), C-reactive protein (116.15 vs. 105.50; *p* = 0.007), and interleukin-6 (17.24 vs. 9.39; *p* = 0.027). A documented sepsis diagnosis was more frequent in the culture-positive group (48.3% vs. 28.1%; OR 2.39, 95% CI 1.47–3.88; *p* < 0.001). Mortality was numerically higher with culture positivity (20.2% vs. 14.4%) but did not reach statistical significance (*p* = 0.188). In the adjusted model, higher leukocyte count remained independently associated with culture positivity (aOR 1.10 per 1 × 10^9^/L, 95% CI 1.05–1.15; *p* < 0.001), while vaccination status showed an inverse exploratory association (aOR 0.58, 95% CI 0.35–0.96; *p* = 0.033). The exploratory model AUC was 0.672 (bootstrap 95% CI 0.608–0.731); calibration was not assessed. Blood-culture positivity was common in this hospitalized BA.5 cohort and showed a nearly balanced Gram-positive/Gram-negative distribution. Leukocytosis and clinically documented sepsis were the clearest correlates of positivity, whereas mortality did not differ significantly. These findings support selective blood-culture use driven by bacterial infection signals and emphasize the need to connect antimicrobial decisions to microbiological evidence.

## 1. Introduction

Bacterial infection has been a persistent diagnostic challenge throughout the COVID-19 pandemic. Patients hospitalized with SARS-CoV-2 infection frequently present with fever, hypoxemia, leukocyte abnormalities, and elevated inflammatory biomarkers, all of which can overlap with the clinical phenotype of bacterial sepsis. This diagnostic uncertainty contributed to extensive blood-culture sampling and widespread empirical antibiotic use, particularly during the early pandemic [1,2,3,4].

The clinical impact of COVID-19 extends beyond infectious diseases and critical care to specialty-specific care pathways and vulnerable populations. In obstetrics, Amadori et al. retrospectively compared 294 deliveries from a prepandemic period with 256 deliveries during and immediately after the COVID-19 lockdown and reported fewer hospitalizations during pregnancy (3.9% vs. 8.9%; *p* = 0.004) together with a higher induction-of-labor rate (35.9% vs. 23.9%; *p* = 0.002), while antepartum and intrapartum complication rates were not significantly different [5]. Severe maternal COVID-19 could also require complex multidisciplinary decision-making when respiratory failure developed and evidence-based pregnancy-specific protocols were limited [6]. These cross-specialty observations are relevant to the present study because healthcare utilization, admission thresholds, case mix, and diagnostic intensity can alter the clinical context in which blood cultures are obtained and, therefore, influence observed culture positivity and antimicrobial exposure.

Early studies nevertheless showed that microbiologically confirmed bacterial coinfection at hospital presentation was substantially less common than antibiotic exposure. Sepulveda et al. reported bacteremia in 3.8% of patients with COVID-19, falling to 1.6% after exclusion of likely skin contaminants, while Yu et al. similarly described a low rate of clinically relevant bloodstream infection together with a substantial burden of contaminated cultures [1,2]. Meta-analytic evidence subsequently estimated bloodstream infection (BSI) rates at approximately 7% of hospitalized patients overall, with much higher rates among critically ill patients and those exposed to invasive devices, prolonged hospitalization, and broad-spectrum antimicrobials [7,8,9,10,11].

The epidemiology of bloodstream infection in COVID-19 is therefore strongly dependent on when cultures are obtained and on the clinical setting. Community-onset bacterial coinfection appears uncommon, whereas secondary and hospital-acquired infections become increasingly relevant with critical illness, central venous catheterization, mechanical ventilation, immunomodulatory treatment, and prolonged intensive-care exposure [8,9,10,11,12,13,14]. At the same time, antimicrobial stewardship remains essential because indiscriminate antibiotic use can select resistant organisms without improving outcomes when bacterial infection is absent [15,16,17,18].

Most of the foundational literature was generated during the ancestral, Alpha, or Delta periods. Data specifically describing blood cultures during the Omicron BA.5 wave are limited, even though vaccination coverage, prior immunity, therapeutic strategies, and hospitalization patterns had changed substantially by mid-2022. A temporally homogeneous BA.5 cohort may therefore provide useful insight into the microbiological spectrum and clinical signals associated with blood-culture positivity in a later pandemic phase. To our knowledge, few studies have evaluated, within the same BA.5 cohort, culture positivity, organism distribution, admission inflammatory markers, documented sepsis, antimicrobial exposure, and short-term outcomes as an integrated clinical-microbiological phenotype [19]. This combined perspective represents the principal novelty of the present study.

The aim of the present study was to determine the frequency and microbiological distribution of positive blood cultures among patients hospitalized with SARS-CoV-2 infection during the Omicron BA.5 wave and to compare demographic, clinical, inflammatory, antimicrobial-treatment, and short-term outcome characteristics between patients with positive cultures and those with no growth. A secondary objective was to explore independent correlates of blood-culture positivity using a parsimonious multivariable model.

## 2. Materials and Methods

### 2.1. Study Design, Setting, and Reporting Guideline

We conducted a retrospective observational cohort study of consecutive patients hospitalized with confirmed SARS-CoV-2 infection during the sixth pandemic wave represented in the source database, corresponding to 12 July–25 September 2022 and the Omicron BA.5 period. The analytic cohort comprised 395 patients with an available blood-culture result. The study was performed at the Clinical Hospital of Infectious Diseases and Pneumophysiology “Dr. Victor Babeș” Timișoara, Romania.

The study is reported in accordance with the Strengthening the Reporting of Observational Studies in Epidemiology (STROBE) statement for cohort studies, an EQUATOR Network reporting guideline [20].

### 2.2. Eligibility, Recruitment, and Sample-Size Considerations

**Figure 1:**
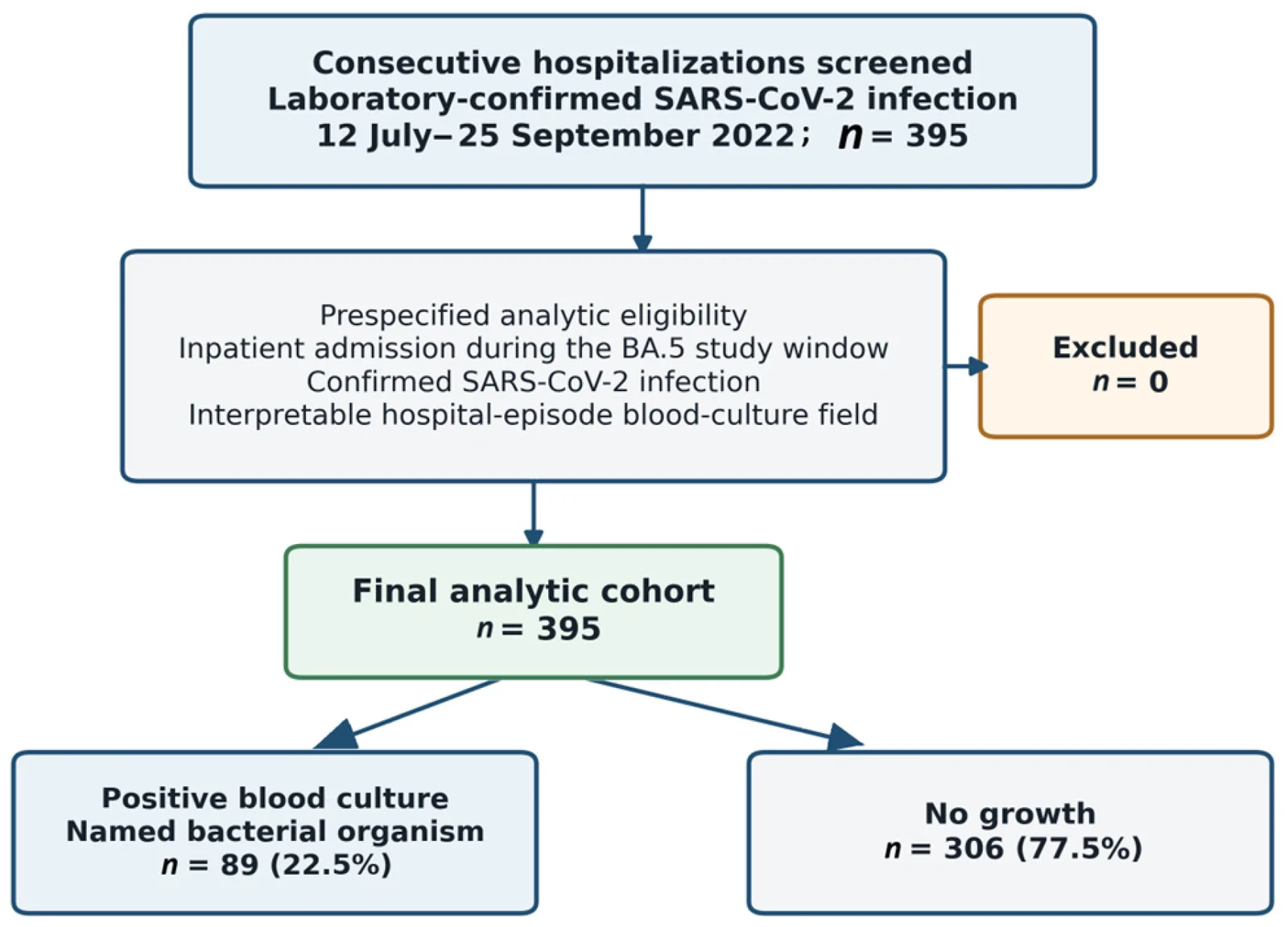
Retrospective cohort recruitment and analysis flow. The fixed-period source cohort comprised 395 consecutive hospitalizations; all records met the prespecified clinical, temporal, and data-availability criteria, so no record was excluded. Culture-positive and no-growth categories were mutually exclusive and based on the recorded hospital-episode blood-culture field. Blue boxes represent the screening, eligibility assessment, and blood-culture outcome categories; the orange box indicates excluded patients; and the green box denotes the final analytic cohort.

Inclusion criteria were: (i) inpatient hospitalization at the study center during 12 July–25 September 2022; (ii) laboratory-confirmed SARS-CoV-2 infection; and (iii) a hospital-episode blood-culture field that could be classified as either a named bacterial organism or “No growth”. Exclusion criteria were outpatient-only encounters, admissions outside the prespecified interval, absence of confirmed SARS-CoV-2 infection, duplicate records from the same hospitalization, and missing, ambiguous, or otherwise uninterpretable blood-culture documentation. The source extract contained 395 consecutive records; all satisfied the prespecified criteria and entered the analysis (Figure 1). No restrictions were applied according to sex, residence, vaccination status, or comorbidity.

The sample size was determined by a census of all consecutive eligible records within the fixed study window rather than by an a priori power calculation. The final cohort comprised 395 patients, including 89 blood-culture-positive events. With five prespecified predictor parameters in the main logistic model, this corresponded to 17.8 outcome events per parameter and supported the deliberately parsimonious exploratory analysis. The cohort was not designed to establish equivalence for mortality or other secondary outcomes; therefore, non-significant comparisons were interpreted together with their effect estimates and 95% confidence intervals.

### 2.3. Blood-Culture Definition and Microbiological Variables

The primary endpoint was blood-culture positivity. Patients were classified as culture positive when the database recorded a named bacterial species and as culture negative when the result was documented as “No growth”. The available dataset did not include the number of culture sets, collection time, bottle-level results, time to positivity, or a formal adjudication of contamination. Accordingly, the term “blood-culture positivity” is used throughout the manuscript and should not be interpreted as synonymous with a prospectively adjudicated BSI endpoint.

Recorded isolates were grouped as Gram-positive or Gram-negative organisms. Species-level antimicrobial susceptibility fields in the source workbook were not used to infer patient-specific resistance because the encoded patterns were not sufficiently granular to establish that they represented individual isolate antibiograms. Detailed information on the blood-culture system, number and volume of sets, organism-identification method, and antimicrobial susceptibility testing (AST) standard/platform was not captured in the analytic dataset and therefore could not be reconstructed reliably for this retrospective analysis.

### 2.4. Clinical and Laboratory Variables

Variables evaluated included age, sex, urban/rural residence, vaccination status, body mass index (BMI), smoking status, chronic obstructive pulmonary disease (COPD), diabetes mellitus, oxygen requirement, antiviral and corticosteroid treatment, antibiotic exposure, admission oxygen saturation (SpO_2_), leukocyte count, erythrocyte sedimentation rate (ESR), D-dimer, C-reactive protein (CRP), interleukin-6 (IL-6), procalcitonin, ferritin, urea, creatinine, length of hospital stay, and in-hospital outcome.

Vaccination was dichotomized as any recorded COVID-19 vaccination versus unvaccinated. Diabetes was defined by the presence of type 1, type 2, or newly diagnosed diabetes in the available fields. Antibiotic exposure was defined as any documented systemic antibiotic treatment during the hospitalization. Because the database did not encode antibiotic timing relative to blood-culture collection, antibiotic exposure was analyzed as an associated treatment variable and not as a predictor that preceded culture positivity.

A sepsis variable was derived conservatively from an explicit documentation of the term “sepsis” in the infectious-complications field. In-hospital mortality was derived from the discharge-evolution field, which consistently distinguished favorable outcome from death. Variables with internally inconsistent non-binary coding in the source spreadsheet, including the dedicated ICU and cardiorespiratory-arrest columns, were not used for inferential analyses.

### 2.5. Statistical Analysis

Continuous variables are summarized as median and interquartile range (IQR), and categorical variables as number and percentage. Between-group comparisons were performed with the Mann–Whitney U test for continuous variables and Fisher’s exact test or Pearson’s chi-square test for categorical variables, according to cell counts. Odds ratios (ORs) with 95% confidence intervals (CIs) were calculated for selected categorical associations.

A parsimonious multivariable logistic regression model was used to explore independent correlates of blood-culture positivity. To reduce overfitting and limit sensitivity to extreme inflammatory values, the prespecified main model included age (per 10-year increase), male sex, vaccination status (any vaccination vs. unvaccinated), BMI (per 5 kg/m^2^ increase), and admission leukocyte count (per 1 × 10^9^/L increase). Model discrimination was summarized by the area under the receiver-operating-characteristic curve (AUC), with a bootstrap 95% CI. Because the model was prespecified as an exploratory association model rather than a clinical prediction model, formal calibration assessment was not prespecified; specifically, no Hosmer-Lemeshow goodness-of-fit test or calibration plot was performed. This limitation is reported explicitly, and the model is not interpreted as providing individual risk estimates or clinical decision thresholds. Two-sided *p* values < 0.05 were considered statistically significant.

### 2.6. Missing Data and Potential Sources of Bias

Blood-culture classification and all variables displayed in Table 1 were complete for all 395 participants. The outcome and all five covariates included in the multivariable model were also complete; therefore, the model included the full analytic cohort (*n* = 395; 89 positive events). No missing values were imputed, and no participant was excluded from the multivariable analysis because of missing data. Variables with internally inconsistent coding were excluded from inferential analysis before model fitting. Selection bias was reduced by using consecutive hospitalizations from a fixed period and prespecified eligibility criteria without outcome-driven exclusions. Nevertheless, because an interpretable blood-culture result was required, the cohort represents culture-assessed hospitalized patients and may be enriched for clinical suspicion of bacterial infection relative to all BA.5 hospitalizations.

Information and misclassification bias were limited by prespecified operational definitions, but could not be eliminated because collection timing, number of sets, bottle-level results, time to positivity, and formal contamination adjudication were unavailable. Confounding was addressed with a small prespecified covariate set; residual confounding by disease severity, invasive-device exposure, immunosuppression, and timing within the hospital stay remained possible and was considered when interpreting the associations.

### 2.7. Ethics

The study was conducted in accordance with the Declaration of Helsinki and approved by the Ethics Committee of the Clinical Hospital of Infectious Diseases and Pneumophysiology “Dr. Victor Babeș” Timișoara, Romania (approval no. 4711/20 May 2026). The approval covered the retrospective analysis and scientific publication of anonymized clinical data routinely collected between 1 March 2020 and 31 December 2024. Written informed consent was obtained from all subjects involved in the study and included permission to use anonymized clinical data for research and publication.

## 3. Results

### 3.1. Cohort Characteristics and Blood-Culture Yield

The study included 395 hospitalized patients with SARS-CoV-2 infection and an available blood-culture result. Median age was 72.0 years (IQR 65.23–79.05), 213 patients (53.9%) were male, and 264 (66.8%) had received at least one recorded COVID-19 vaccine regimen. Median BMI was 28.2 kg/m^2^ (IQR 23.7–32.4). Overall, 285 patients (72.2%) received systemic antibiotic therapy during hospitalization and 62 (15.7%) died in hospital.

**Figure 2:**
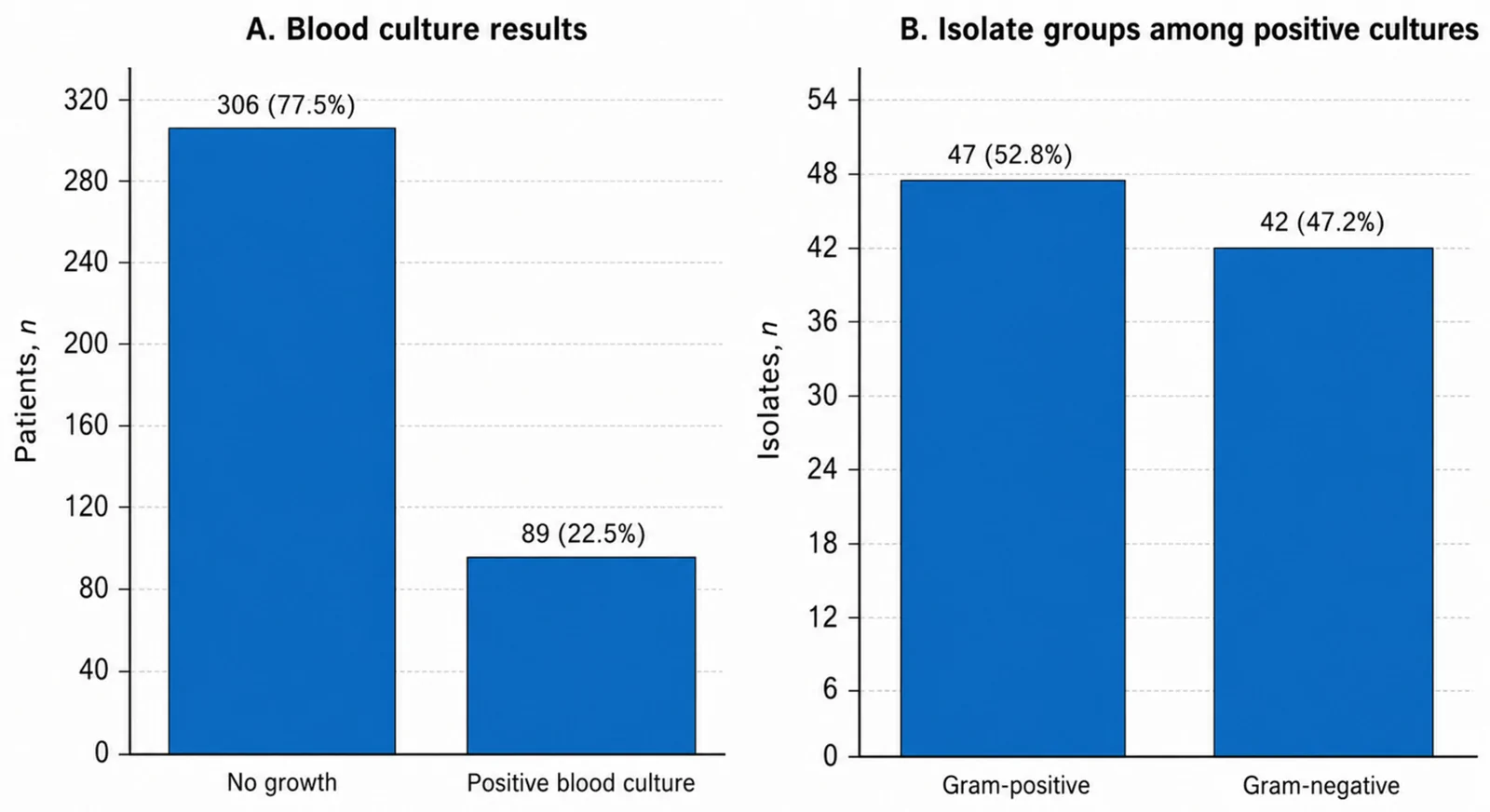
Blood-culture results in the study cohort. (**A**) Distribution of blood-culture results among the 395 patients included in the analysis: 306 patients (77.5%) had no growth and 89 patients (22.5%) had a blood culture positive for a named bacterial organism. (**B**) Distribution of the 89 recorded isolates by Gram stain group: 47 isolates (52.8%) were Gram positive and 42 isolates (47.2%) were Gram negative. Percentages in panel (**A**) use the full analytic cohort as the denominator, whereas percentages in panel (**B**) use positive blood cultures as the denominator.

Blood cultures showed no growth in 306/395 patients (77.5%) and were positive for a named bacterial organism in 89/395 (22.5%), as shown in Figure 2. The culture-positive and no-growth groups were broadly similar in age, sex, BMI, COPD, diabetes, SpO_2_, and length of stay (Table 1).

### 3.2. Microbiological Spectrum

Among the 89 positive blood cultures, 47 isolates (52.8%) were Gram positive and 42 (47.2%) were Gram negative. *Streptococcus pneumoniae* was the most frequently recorded organism (18/89; 20.2%), followed by *Enterococcus faecalis* and *Klebsiella pneumoniae* (16/89 each; 18.0%), and by *Staphylococcus aureus*, *Escherichia coli*, and *Pseudomonas aeruginosa* (13/89 each; 14.6%) (Table 2).

Mortality was 21.3% among patients with Gram-positive cultures and 19.0% among those with Gram-negative cultures; this difference was not statistically significant (Fisher’s exact *p* = 1.000). Because isolate-level source attribution and contamination adjudication were unavailable, pathogen-specific outcome percentages are presented descriptively and should not be interpreted as causal organism effects.

### 3.3. Inflammatory Correlates, Sepsis, and Antimicrobial Exposure

**Figure 3:**
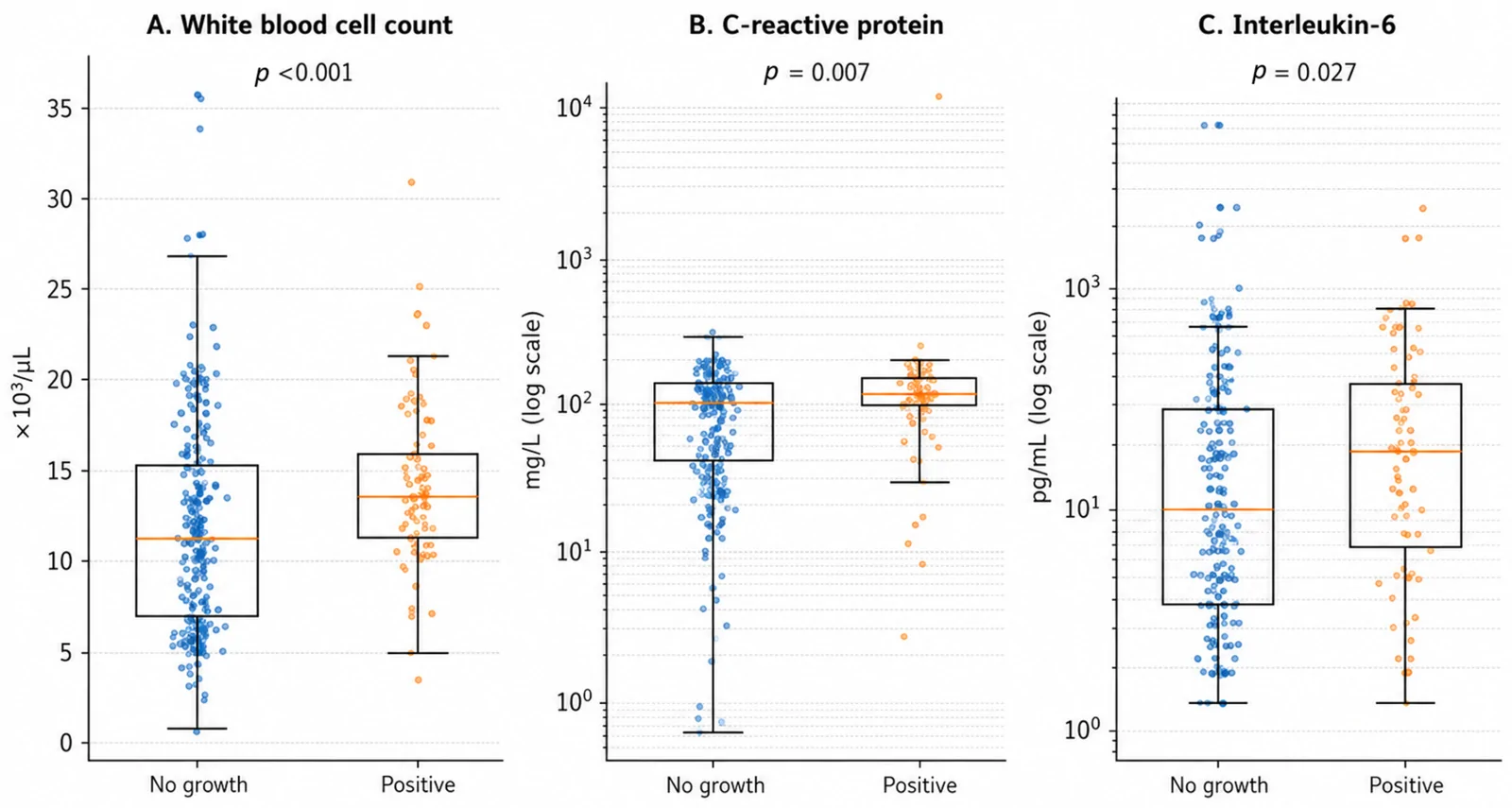
Admission inflammatory markers according to blood-culture result. Box-and-whisker plots with overlaid individual observations compare patients with no growth (*n* = 306) and patients with a positive blood culture (*n* = 89) for (**A**) white blood cell count, (**B**) C-reactive protein and (**C**) interleukin-6. Boxes indicate the median and interquartile range, whiskers extend to 1.5 times the interquartile range, and each point represents one patient. C-reactive protein and interleukin-6 are displayed on a logarithmic scale because of their strongly right-skewed distributions. *p* values are derived from the Mann–Whitney U test.

Patients with positive cultures had a substantially higher admission leukocyte count than those with no growth (median 13.42 vs. 11.22 × 10^9^/L; *p* < 0.001). CRP (116.15 vs. 105.50; *p* = 0.007) and IL-6 (17.24 vs. 9.39; *p* = 0.027) were also higher in the culture-positive group, as in Figure 3. In contrast, procalcitonin, ESR, D-dimer, ferritin, renal markers, and admission SpO_2_ did not differ significantly between groups.

An explicitly documented sepsis diagnosis occurred in 43/89 culture-positive patients (48.3%) compared with 86/306 patients with no growth (28.1%), corresponding to an unadjusted OR of 2.39 (95% CI 1.47–3.88; *p* < 0.001). All culture-positive patients received antibiotic therapy, compared with 196/306 (64.1%) of patients with no growth (*p* < 0.001). The temporal relationship between culture sampling and antibiotic initiation was unavailable; therefore, this comparison describes treatment exposure rather than culture-directed prescribing.

In-hospital mortality occurred in 18/89 culture-positive patients (20.2%) and 44/306 patients with no growth (14.4%). The unadjusted OR for death was 1.51 (95% CI 0.82–2.77), but the difference was not statistically significant (*p* = 0.188). Median length of stay was similarly not different between groups (10 vs. 9 days; *p* = 0.742).

### 3.4. Multivariable Analysis of Blood-Culture Positivity

**Figure 4:**
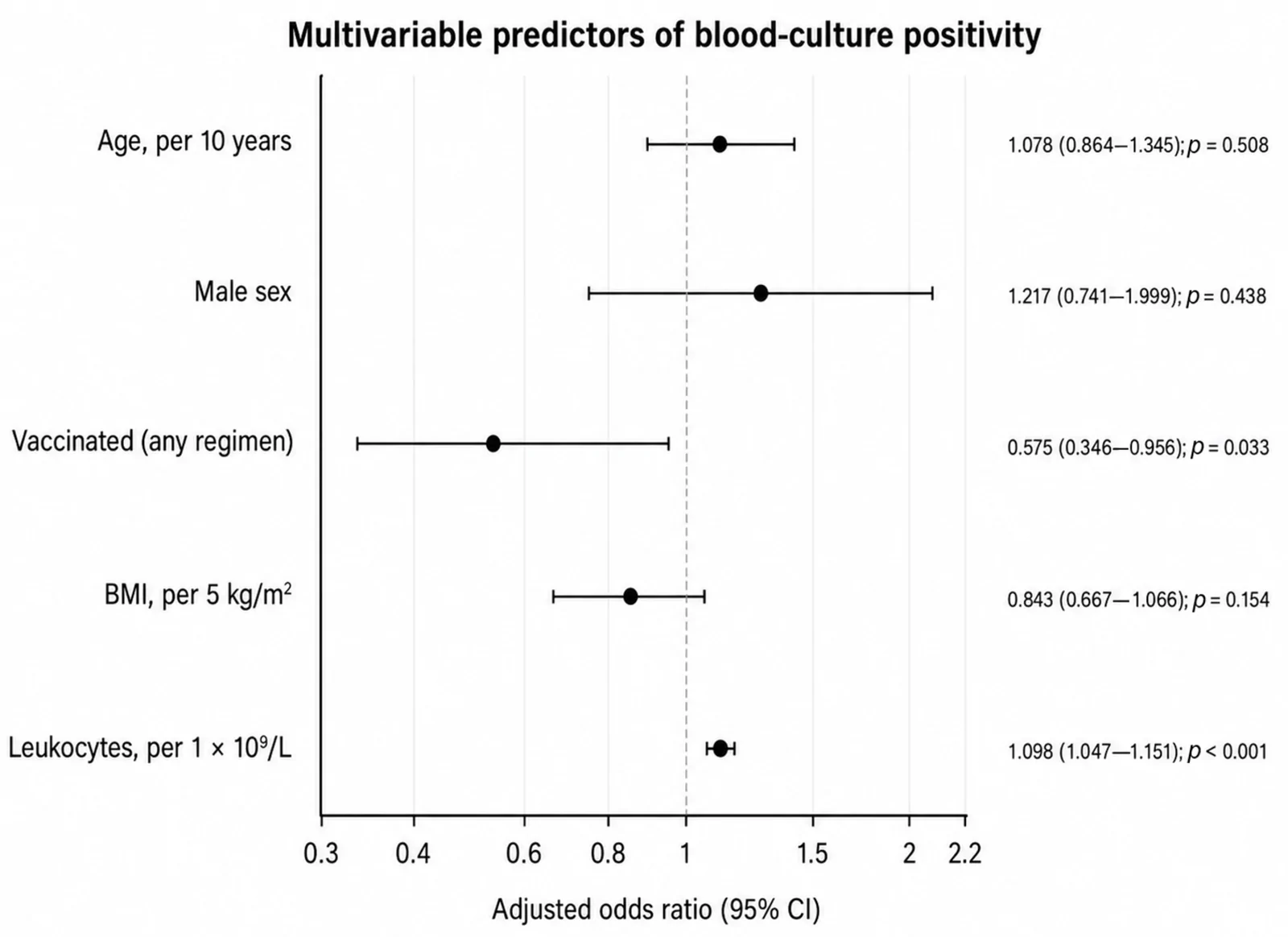
Forest plot of the multivariable logistic regression model for blood-culture positivity. Points represent adjusted odds ratios and horizontal lines the corresponding 95% confidence intervals; the vertical dashed line marks the null value (odds ratio = 1). The *x*-axis is displayed on a logarithmic scale. Continuous variables are scaled as indicated (age per 10 years, body mass index per 5 kg/m^2^, and leukocytes per 1 × 10^9^/L). Higher admission leukocyte count was independently associated with blood-culture positivity, whereas vaccination status showed an inverse exploratory association; age, sex and body mass index were not independently associated with the outcome.

The multivariable model included all 395 participants (89 positive events). Admission leukocyte count remained independently associated with blood-culture positivity (aOR 1.10 per 1 × 10^9^/L increase, 95% CI 1.05–1.15; *p* < 0.001). Vaccination status showed an inverse association with positivity (aOR 0.58, 95% CI 0.35–0.96; *p* = 0.033), whereas age, sex, and BMI were not independently associated. Discrimination was modest (AUC 0.672; bootstrap 95% CI 0.608–0.731) (Table 3 and Figure 4). No formal calibration statistic, including the Hosmer-Lemeshow test, or calibration plot was generated because prediction was not the purpose of the model. Accordingly, these estimates should be interpreted only as exploratory adjusted associations, not as an individual risk-prediction tool. The vaccination finding is non-causal because residual confounding by health status, prior infection, admission phenotype, and healthcare exposure cannot be excluded.

## 4. Discussion

### 4.1. Principal Findings and Novelty

In this temporally homogeneous cohort of 395 patients hospitalized during the Omicron BA.5 wave, 89 (22.5%) had a blood culture positive for a named bacterial organism. Gram-positive and Gram-negative isolates were nearly balanced. Culture positivity was associated with higher admission leukocyte count, C-reactive protein (CRP), interleukin-6 (IL-6), and more frequent clinical documentation of sepsis; leukocyte count remained independently associated after adjustment. Mortality was numerically higher in culture-positive patients but did not differ significantly. The principal novelty is the integrated assessment of culture yield, organism distribution, inflammatory phenotype, sepsis documentation, antimicrobial exposure, and short-term outcomes within a single BA.5 hospitalization window, thereby reducing the variant and treatment heterogeneity inherent to multi-wave cohorts [19].

### 4.2. Blood-Culture Yield and Microbiological Context

The observed positivity of 22.5% exceeds bacteremia yields reported in several early-pandemic cohorts [1,2] and pooled estimates of approximately 7–8% among hospitalized patients [7,19]. This difference should be interpreted in the context of a culture-assessed inpatient cohort rather than as population incidence. Secondary bloodstream infection is enriched by critical illness, mechanical ventilation, central venous access, immunomodulation, and prolonged hospitalization [8,9,10,11,12,13,14,21,22,23,24,25], while changes in sampling thresholds and case mix can also alter observed yield [26,27,28].

The organism distribution was clinically plausible for a mixed hospitalized population: *Streptococcus pneumoniae* was most frequent, followed by *Enterococcus faecalis*, *Klebsiella pneumoniae*, *Staphylococcus aureus*, *Escherichia coli*, and *Pseudomonas aeruginosa* (Table 2). The near parity between Gram-positive and Gram-negative organisms differs from the Gram-negative predominance reported in a Romanian intensive-care cohort [29] but resembles later single-center observations with substantial Gram-positive representation [30]. These differences support unit-level microbiological surveillance rather than a universal COVID-19 pathogen profile; isolate-level resistance was not analyzed because patient-specific susceptibility data could not be validated [31,32].

### 4.3. Inflammatory Correlates, Sepsis, and Model Performance

Admission leukocyte count showed the clearest association with culture positivity and remained independently associated after adjustment (aOR 1.10 per 1 × 10^9^/L, 95% CI 1.05–1.15). CRP and IL-6 were also higher in unadjusted comparisons, but their distributions overlapped substantially, consistent with the nonspecific inflammatory response of severe SARS-CoV-2 infection [33,34,35,36]. Procalcitonin did not differ significantly and should not be used as a stand-alone trigger for antibacterial treatment [16,17]. Clinically documented sepsis was more frequent with a positive culture (48.3% vs. 28.1%; OR 2.39, 95% CI 1.47–3.88), although this association does not establish that the cultured organism caused organ dysfunction because culture-negative sepsis is common and COVID-19 itself may satisfy Sepsis-3 criteria [37,38].

The adjusted model showed only modest discrimination (AUC 0.672; bootstrap 95% CI 0.608–0.731). Formal calibration was not assessed, including by Hosmer-Lemeshow testing or calibration plotting, because the model was designed for exploratory association estimation rather than prediction. The absence of calibration assessment, together with the modest AUC and lack of external validation, precludes bedside predictive use. The inverse vaccination association should likewise be considered hypothesis-generating because residual confounding cannot be excluded.

### 4.4. Antimicrobial Stewardship and Outcomes

Antimicrobial exposure substantially exceeded microbiological confirmation: all culture-positive patients and 64.1% of those with no growth received antibiotics, corresponding to 72.2% of the cohort. Although a sterile blood culture does not exclude non-bacteremic infection and prescribing indications were unavailable, this gap identifies a stewardship opportunity consistent with broader COVID-19 evidence [4,15,18,19,39]. Blood cultures should be obtained when bacterial infection is clinically plausible, and empirical therapy should be reassessed after 48–72 h using microbiology, clinical trajectory, imaging, and device status; infection-prevention measures and rapid diagnostics may further reduce unnecessary exposure [40,41].

In-hospital mortality was 20.2% in culture-positive patients and 14.4% in those with no growth (OR 1.51, 95% CI 0.82–2.77; *p* = 0.188), while length of stay was similar. The non-significant mortality result should not be interpreted as evidence of no prognostic effect: only 18 deaths occurred in the positive group, and the exposure combined organisms of differing virulence with uncertain contamination status and unknown sampling time [19,21,22,42].

### 4.5. Strengths, Limitations, and Potential Bias

Strengths include the single-wave BA.5 design, consecutive records from a fixed interval, complete blood-culture classification, complete data for all variables reported in Table 1 and all multivariable-model covariates, and integration of microbiological and clinical domains. Prespecified operational definitions and conservative terminology also reduced overinterpretation of positive cultures as adjudicated bloodstream infection.

The principal limitations are the retrospective, single-center design and restriction to culture-assessed patients, which may enrich the cohort for suspected bacterial infection and limit generalizability. Culture timing, number and volume of sets, bottle-level positivity, time to positivity, infection source, device exposure, contamination adjudication, antibiotic timing, and prior outpatient antibiotic exposure were unavailable; consequently, community-onset and hospital-acquired events could not be reliably distinguished. Sepsis relied on explicit documentation, BA.5 attribution was calendar-based rather than sequencing-confirmed, and residual confounding by severity, intensive-care exposure, immunosuppression, and invasive devices remains possible.

Although 89 positive events supported the five-parameter exploratory model, its modest discrimination, absence of formal calibration assessment and external validation, and lack of multiplicity adjustment preclude predictive interpretation. Precision was also limited for mortality and organism-specific outcomes, and follow-up ended at discharge.

### 4.6. Clinical Implications, Cross-Specialty Context, and Future Research

These findings support selective blood-culture acquisition when bacterial infection is clinically plausible and prompt antimicrobial reassessment when microbiological results become available. Leukocytosis may strengthen clinical suspicion, but neither a single inflammatory marker nor the present exploratory model is sufficiently discriminative to replace bedside assessment. Empirical therapy should remain aligned with local epidemiology and resistance patterns.

Cross-specialty interpretation also requires caution. Obstetric cohorts illustrate this point: pandemic-related changes in antenatal hospitalization and labor induction occurred even without a parallel increase in major peripartum complications [5]. Specialty-specific admission thresholds, baseline physiology, invasive-device exposure, antimicrobial indications, and tolerance for diagnostic uncertainty differ substantially. Consequently, the 22.5% culture positivity observed here should not be generalized as a universal COVID-19 rate; it is a finding from a culture-assessed infectious-diseases inpatient cohort hospitalized during a defined Omicron BA.5 period.

Prospective multicenter studies should capture culture indication and timing, number and volume of sets, bottle-level results, time to positivity, infection source, device exposure, validated severity measures, and isolate-level susceptibility, with formal calibration and external validation if prediction models are developed. Patient-level variant confirmation and post-discharge follow-up would further help distinguish community coinfection from secondary hospital-acquired bloodstream infection and clarify the mortality and vaccination signals observed here.

## 5. Conclusions

In this culture-assessed 395-patient cohort hospitalized during the SARS-CoV-2 Omicron BA.5 wave, 22.5% had a blood culture positive for a named bacterial organism. Gram-positive and Gram-negative isolates occurred in nearly equal proportions, with *S. pneumoniae*, *E. faecalis*, *K. pneumoniae*, *S. aureus*, *E. coli*, and *P. aeruginosa* comprising the recorded microbiological spectrum. Blood-culture positivity was associated with higher leukocyte counts, CRP and IL-6 levels, and a greater frequency of clinically documented sepsis, while the observed increase in mortality did not reach statistical significance. The findings support selective culture acquisition when bacterial infection is clinically plausible and rapid antimicrobial reassessment once microbiological data become available. Prospective Omicron-era studies incorporating culture timing, source adjudication, device exposure, time to positivity, and patient-level susceptibility data are needed to distinguish community coinfection from secondary hospital-acquired BSI and to optimize antimicrobial stewardship.

## Figures and Tables

**Table 1:** Clinical and laboratory characteristics according to blood-culture result.

Variable	Positive (*n* = 89)	No Growth (*n* = 306)	*p* Value
Age, years	72.86 (65.79–80.03)	72.00 (65.15–79.00)	0.442
Male sex	51 (57.3)	162 (52.9)	0.546
Vaccinated (any regimen)	52 (58.4)	212 (69.3)	0.073
BMI, kg/m^2^	26.98 (23.40–31.20)	28.55 (24.23–32.50)	0.111
COPD	9 (10.1)	43 (14.1)	0.378
Diabetes mellitus	16 (18.0)	60 (19.6)	0.879
Admission SpO_2_, %	90.94 (89.05–92.23)	91.00 (89.00–92.95)	0.912
Leukocytes, ×10^9^/L	13.42 (11.28–15.79)	11.22 (7.09–15.12)	<0.001
CRP	116.15 (97.29–150.59)	105.50 (40.01–139.66)	0.007
IL-6	17.24 (5.20–37.45)	9.39 (3.80–28.54)	0.027
Procalcitonin	0.50 (0.20–0.90)	0.39 (0.14–0.90)	0.147
Antibiotic exposure	89 (100.0)	196 (64.1)	<0.001
Documented sepsis	43 (48.3)	86 (28.1)	<0.001
Length of stay, days	10 (5–19)	9 (7–14)	0.742
In-hospital mortality	18 (20.2)	44 (14.4)	0.188

Data are median (IQR) or *n* (%). All displayed variables were complete for all 395 participants. BMI, body mass index; COPD, chronic obstructive pulmonary disease; SpO_2_, peripheral oxygen saturation; CRP, C-reactive protein; IL-6, interleukin-6.

**Table 2:** Distribution of organisms and descriptive outcomes.

Organism	*n*	% Positive	% Cohort	Mortality *n* (%)	Sepsis *n* (%)
*Streptococcus pneumoniae*	18	20.2	4.6	4 (22.2)	6 (33.3)
*Enterococcus faecalis*	16	18.0	4.1	5 (31.2)	9 (56.2)
*Klebsiella pneumoniae*	16	18.0	4.1	1 (6.2)	10 (62.5)
*Staphylococcus aureus*	13	14.6	3.3	1 (7.7)	6 (46.2)
*Escherichia coli*	13	14.6	3.3	4 (30.8)	4 (30.8)
*Pseudomonas aeruginosa*	13	14.6	3.3	3 (23.1)	8 (61.5)
All positive cultures	89	100.0	22.5	18 (20.2)	43 (48.3)
No growth	306	-	77.5	44 (14.4)	86 (28.1)

**Table 3:** Multivariable logistic regression for blood-culture positivity.

Variable	Adjusted OR	95% CI	*p* Value
Age, per 10 years	1.078	0.864–1.345	0.508
Male sex	1.217	0.741–1.999	0.438
Vaccinated (any regimen)	0.575	0.346–0.956	0.033
BMI, per 5 kg/m^2^	0.843	0.667–1.066	0.154
Leukocytes, per 1 × 10^9^/L	1.098	1.047–1.151	<0.001

Model *n* = 395 (89 positive events); no model covariate or outcome data were missing. AUC = 0.672; bootstrap 95% CI 0.608–0.731. Formal calibration was not assessed (no Hosmer-Lemeshow test or calibration plot) because the model was exploratory and was not developed as a clinical prediction rule. OR, odds ratio; CI, confidence interval; BMI, body mass index.

## Data Availability

Deidentified clinical and laboratory data supporting the findings are available from the corresponding authors upon reasonable request, subject to institutional data-sharing policies and applicable privacy requirements.

## Abbreviations

The following abbreviations are used in this manuscript:

aOR	adjusted odds ratio
AST	antimicrobial susceptibility testing
AUC	area under the receiver-operating-characteristic curve
BMI	body mass index
BSI	bloodstream infection
CI	confidence interval
COPD	chronic obstructive pulmonary disease
COVID-19	coronavirus disease 2019
CRP	C-reactive protein
ESR	erythrocyte sedimentation rate
ICU	intensive care unit
IL-6	interleukin-6
IQR	interquartile range
ISARIC	International Severe Acute Respiratory and Emerging Infection Consortium
OR	odds ratio
SARS-CoV-2	severe acute respiratory syndrome coronavirus 2
SpO2	peripheral oxygen saturation
STROBE	Strengthening the Reporting of Observational Studies in Epidemiology
WHO	World Health Organization
